# To What Extent Is Long-term Care Representative of Elderly Care? A Case Study of Elderly Care Financing in Lombardy, Italy

**DOI:** 10.15171/ijhpm.2017.22

**Published:** 2017-02-25

**Authors:** Elenka Brenna, Lara Gitto

**Affiliations:** ^1^Department of Economics and Finance, Università Cattolica del Sacro Cuore, Milan, Italy.; ^2^CEIS EEHTA (Economic Evaluation & Health Technology Assessment), Università di Roma “Tor Vergata,” Roma, Italy.

**Keywords:** Long-term Care (LTC) Services, Elderly Care Financing, European LTC Policies

## Abstract

The ageing of European population has been rapidly increasing during the last decades, and the problem of elderly care financing has become an issue for policy-makers. Long-term care (LTC) financing is considered a suitable proxy of the resources committed to elderly care by each government, but the preciseness of this approximation depends on the extent to which LTC is representative of elderly care within each country. Since there is a broad heterogeneity in LTC funding, organization and setting among European States, it is difficult to find a common parameter representing the public resources destined to the elderly care. We address these topics employing as a case study an Italian region, Lombardy, which in terms of population, dimension, healthcare organization and economic development could be compared to other European countries. The method we suggest, which consists basically in a careful estimate of all the public resources employed in the provision of services exclusively destined to the elderly, could be applied, with the due differences, to other European countries or regions.

## Introduction

The term long-term care (LTC) is broadly applied to define “*a range of services and assistance for people who depend on help with daily living activities and/or are in need of some permanent nursing care.*”^1^ In Europe (EU 28), people in need of some permanent care are mostly represented by the share of older population^ [1]^ for two reasons: first, the ageing of European population has been rapidly increasing during the last decades, second, older persons, especially over 75 years of age, are more likely to develop chronic pathologies, comorbidities or other impairing diseases, that require continuous assistance.^2^

In Europe, the share of people over 65 reached in 2015 18.9%, with an increase of 2.3% compared with 10 years earlier. Italy, Germany, and Greece report respectively a value of 21.7%, 21% and 20.9%, while Ireland shows the lowest percentage, 13.0%.^3^

The way elderly formal care is organized and financed has then become an issue of increasing weight for policy-makers, also because, at a societal level, it interferes directly with families and individuals’ choices on resources, job and time allocation.^4^

In the comparisons between the Organization for Economic Co-operation and Development (OECD) members or among the European countries, LTC funding is often used as a proxy of the resources dedicated to elderly care.^1,4-8^ On this point, however, two issues need to be addressed. First, LTC services are not targeted *exclusively* to the elderly; they include other categories of recipients, such as people with disabilities or with problems of addiction and, consequently, not all the resources destined to LTC are ultimately devoted to the elderly care. The extent to which LTC services are provided to the elderly depends on the internal organization of each welfare and healthcare system. A second issue concerns the different sources employed to finance LTC services in each country. Some countries include in the LTC financing the cash benefits payed by the social security to the elderly and invalid recipients: in Italy, for example, these benefits account for half of the whole amount destined to the elderly care. However, in many cases, when observing the international comparisons, social security sources are not included within the LTC financing and this might bias the international ranking.^8^

Although during the last years a growing interest in detecting and including the different sources of LTC funding, as well as in considering the heterogeneity of LTC organization and setting among European countries has been shown,^2,9^ the issue of the public resources specifically destined to the elderly by each member State remains unclear.

Looking at the literature on elderly care, there is a consistent branch that investigates the cultural, religious, and institutional variables influencing the provision and funding of formal and informal care across European countries; family ties, for example, play a crucial role in the assistance provided to the frailest family members, with consequences on the financial load of each member State.^10-13^ However, if we search for a clear definition and assessment of the resources committed by each government exclusively to the elderly care, we just find some studies that refer to specific estimates, such as the expenditure for particular pathologies or services.^14-17^ As a result, the issue of the amount of the public resources actually spent by each country to the elderly LTC is uncertain. The purpose of this debate is to provide a counterpoint to current practices for estimating the cost of elderly care, which may not truly reflect the costs incurred for providing care to those 65+.

We focus on Lombardy region as a case study in order to analyze and compute the exact amount of funding devoted to elderly care in a European region.

The Italian National Health Service (NHS) is a decentralized healthcare system, where regions are responsible for the funding and delivery of healthcare services. In terms of population, dimension, healthcare system and economic development, Lombardy is comparable to several Western European countries^ [2]^ such as Austria, the Netherland, and Belgium.

The method we address, which consists basically in a careful examination of all the public resources employed in the provision of services *exclusively* destined to the elderly, could be applied, with the due differences, to other European countries or regions.

## Financing Long-term Care and Elderly Care in Italy

The Italian LTC financing is managed through three institutional levels: central, regional, and community based.

The Central Government, through social security, administers the so-called indemnities for caring, cash benefits provided to invalid people, 80% of whom are elderly. Checks are granted in relation to the health condition of the recipient and independently from his/her economic position. Indemnities for caring represent almost 50% of the whole elderly care financing. Furthermore, the size of specific flows that transit through the regions and are ultimately devoted to community care, are set at the central level. Some examples are the National Fund for LTC (*Fondo Nazionale per la Non Autosufficienza*) or the National Fund for Social Policies (*Fondo Nazionale Politiche Sociali*). These funds are designed as “bounded resources,” since, basically, regions should transfer them to the community care, without directly employing them.

Regions are responsible for LTC services: the source of funding is the Regional Health Fund, autonomously administered by each region. Almost all the financing destined to LTC is retained by the regions, except for a small share, devoted to the municipalities to run the community based services. These funds are called “autonomous resources” because they are set yearly accordingly to the regional budget.

Bounded and autonomous resources contribute only a small percentage (from 9% to 13%, depending on the region) to the community services managed by the municipalities, the main share being provided by communities’ direct taxation.

## The Lombardy Welfare System

To quantify the exact amount of resources devoted to elderly LTC, we developed a method that employs both regional balance and data on service consumption. Lombardy has been taken as case study, for the following reasons. Despite its “regional” classification, its population reaches 10 million people, very close to the population of some European countries, such as Belgium, which counts with 11 258 434 inhabitants, Sweden (9 747 355) or Austria (8 576 261).^3,18^ Due to the federal setting of the Italian NHS, Lombardy rules autonomously its health and welfare system; the “Lombardy Model of Healthcare,” was implemented in 1997 on a quasi-market setting and is highly recognised in Italy and abroad.^19^ In terms of comparisons with other healthcare systems, another common indicator is the percentage of public expenditure on healthcare, which is 76% for Austria, 77% for Belgium and Italy, and 84% for Sweden.^20^ Looking at the economic variables, per capita income is €35 700 (data 2014), far above the national Italian average of €26 500 and very close to Belgium (€35 900) or Austria’s values (€38 500). All these features address the Lombardy Healthcare System as a solid case study, which is feasible for comparisons to other countries’ health and welfare systems.

Focusing on elderly care, in Lombardy, people over 65 years old represent the 21.6% of population, a value in line with the Italian average and higher than the EU28 share, which is 18.9%.

Figure reports the regional sources and the institutional subjects involved in the elderly care. The Welfare Authority, which is responsible for LTC at the regional level, is kept separate from the Health Authority. The resources for financing the regional services destined to LTC (nursing homes, homecare, etc) are transferred from the Regional Health Fund (almost 9% of its total amount) to the Welfare Fund. Within each Local Health Authority there are specific administrative departments (so-called ASSI), that manage LTC services at the regional level^ [3] ^.

**Figure:**
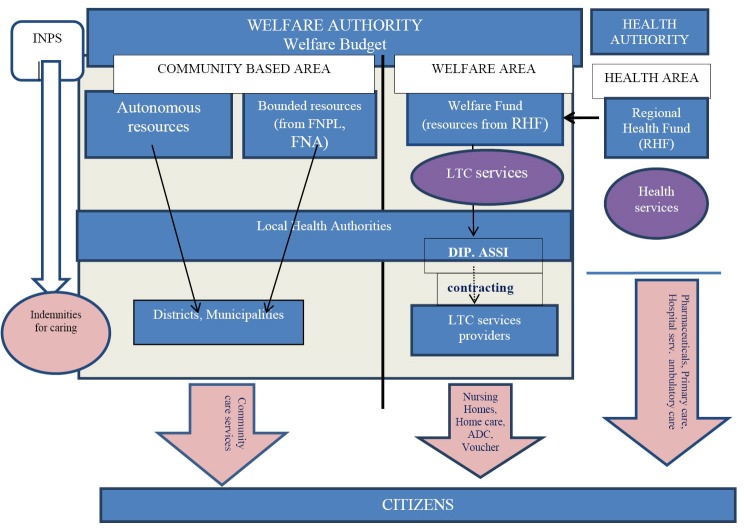
The Welfare Model in Lombardy: Regional Sources, Institutional Subjects, and Resource Allocation. Abbreviations: FNPL, National Fund Social Policies; FNA, National Fund for Dِِisability; INPS, Istituto Nazionale Previdenza Sociale (National Social Security); ASSI, Assistenza Socio Sanitaria Integrata (Internal departments for administering LTC services); LTC, long-term care. Source: Personal elaboration of data drawn from Lombardy Regional Balance^21^ and Report 2015 on National Accounting.^22^

Community based services are directly run by the municipalities; their funding comes mainly from community taxation but there is also a small share (almost 9% of their total amount) originating from bounded and autonomous resources.

### Financing Elderly Care in Lombardy: The Regional Sources

The difficulty in defining the exact amount of the regional resources destined to elderly care is due to the fact that the value of LTC funding as a whole was available, but the resources specifically destined to the elderly care had to be found through the bottom up technique, which requires the identification of every item devoted to elderly care.

For the year 2014, data and information derive from the “Regional Report on Balance indicators.”^23^ In this report, the planned public expenditure is disaggregated into chapters, named “Missions,” which are additionally parted into expenditure items.

Mission 12, named “Social Rights and Policies” includes two programs concerning elderly care: program 3 is specifically addressed to the elderly and devoted to residential buildings’ maintenance (€237 570 in 2014), while program 7 is directed both to the elderly and to other recipients with frailties.

The main amount in the financing of LTC is embodied in Mission 13, which includes the resources devoted to healthcare (namely the Regional Health Fund). As reported above, the Welfare Fund, specifically directed to LTC, absorbs just a small share of the Regional Health Fund, (almost 9% in 2014, for a value of €1 632 000 000). But this amount is not entirely destined to the elderly; for example, in the past legislature, only 63% was dedicated to them.^21^ Applying this percentage, the final amount is €1 028 160 000.

Summing up all the amounts, we were able to find the total funding devoted to the elderly in 2014: €1 052 960 696, corresponding to €490 per resident over 65 (Table 1).

**Table 1:** Regional Expenditure for Elderly Care^a^

	**Denomination**	**Total Expenditure**	**Share for the Elderly**
Mission 12	Social rights and policies	109 881 810	
Program 3	Residential buildings		237 570
Program 7	Planning and management of community services	61 407 814	
		Of which 40% destined to the elderly	24 563 126
Mission 13	Health and healthcare	18 326 395 354	
Program 1 (98.5%)	Financing health services	18 051 499 424	
Health services	Of which 9% devolved to the Regional Social Fund	1 632 000 000	
		Of which 63% destined to the elderly	1 028 160 000
Elderly planned expenditure			1 052 960 696

^a^ Values are in euros. Source: Elaboration of data from Lombardy region, 2014.^23^

### Community-Based Services for the Elderly

The municipalities autonomously run community-based services, which are partly destined to the elderly. Funding comes mainly from municipality’s taxation, while a little share (almost 9%) is collected from regional sources (autonomous and bounded resources).

Municipalities provide the elderly with residential services, home care and cash benefits, for a total financing of €148 million in 2011 (elaboration of data from Istat^24^).

### The Central Level: Indemnities for Caring

In order to complete the analysis of the resources destined to elderly care in Lombardy, we had to compute the value of indemnities for caring, financed by the Central Government and managed through social security programs (see Figure). Indemnities are cash benefits provided to disabled people (elderly in the majority of cases) with the specific aim of furnishing economic help to buy formal care. Indemnities are provided based on the health conditions and independently from the economic position of the user, and are, therefore, considered as an integration on the personal/familiar income.

Our elaboration on Istat data^25^ shows that, in 2014, the indemnities’ expenditure for persons over 65 in Lombardy was €1 183 865 760, which corresponds to almost 50% of the whole amount of resources destined to the elderly in Lombardy.

### Public Financing for Elderly Long-term Care in Lombardy

We were finally able to provide an appropriate estimate of the resources for the elderly LTC in Lombardy. The whole financing for elderly care in 2014 was almost €2.4 billion, €1111 per resident over 65. Indemnities represent half of the amount, regional resources 44% and community based share is 6% (Table 2).

**Table 2:** Public Financing for Elderly LTC in Lombardy – Estimate^a^

	**Absolute Terms**	**Percent**
Regional funds (estimate)	1 052 960 696	44
Community’s funds	148 000 000	6
INPS indemnities	1 183 865 760	50
Tot amount year 2014^a^	2 384 826 456	100
Funding per resident over 65	1111	100

^a^Values are in euros. Abbreviations: INPS, Istituto Nazionale Previdenza Sociale; LTC, long-term care.

In terms of comparisons with other countries, a central issue hangs around the inclusion of the social security funding. If we consider only the LTC elderly services and omit the indemnities, the *per capita* amount would be half, almost €550. For this reason, to avoid bias in the comparisons among countries, it is important to include all the sources of funding, regardless of whether they relate to the health or social services.

## Conclusions

This debate tries to shed some light on the erroneous interpretation which overlaps the concept of “long-term care” with that of “elderly care.”

In fact, LTC services, although mainly directed to the elderly, are also addressed to other kinds of recipients, such as minors or adults with disabilities; consequently, not all the resources destined to LTC are ultimately devoted to the elderly care. Identifying the exact amount of public funding committed by each European government to the elderly assistance, could be of help in the assessment of the policies directed to the ageing population, especially when comparing the health and welfare systems of different EU (European Union) countries. Elderly care is an issue for European societies: caring for an older parent or relative might require the caregiver to quit her job or reduce her leisure time, with possible consequences respectively on families’ income or on the mental/physical health of the caregiver, which may not be prepared to carry this load. This is especially true for those countries where LTC Services are not yet sufficiently developed to absorb the increasing demand of the population ageing.^4,11,12^

Our study defines the organizational scheme of LTC financing for ageing population in Italy. Due to the federalist setting of the Italian Health Service, the analysis is focused on Lombardy, but the method we propose can be applied to other European countries, the rapidity of the results being highly dependent on both data availability and the complexity of the internal welfare system.

At the European level, using a common and well specified indicator capable of clearly defining the public resources specifically destined to the elderly care would help policy-makers in tackling the increasing ageing of the population and, at the same time, avoid possible bias in the international comparisons among countries.

## Acknowledgments

The authors wish to thank two anonymous referees for their valuable and insightful comments which helped to improve the final work.

## Ethical issues

Not applicable.

## Competing interests

Authors declare that they have no competing interests.

## Authors’ contributions

Both authors contributed in an equal measure to the manuscript, that is 50% each.

## Authors’ affiliations

^1^Department of Economics and Finance, Università Cattolica del Sacro Cuore, Milan, Italy. ^2^CEIS EEHTA (Economic Evaluation & Health Technology Assessment), Università di Roma “Tor Vergata,” Roma, Italy.

## Endnotes


[1] According to Eurostat, a person is considered old when she reaches the age of 65 years (http://ec.europa.eu/eurostat/statistics).

[2] Although the statistical values reported refer to the EU 28, the comparisons in terms of healthcare systems are referred to Western European countries, due to their longer permanence within the UE and a common better knowledge of their healthcare and welfare systems.

[3] By the end of 2015 a reform has been implemented in order to unify the Health and Welfare System, but no effects are so far observable on the welfare budget.

